# Predicting Acute and Post-Recovery Outcomes in Cerebral Malaria and Other Comas by Optical Coherence Tomography (OCT in CM) – A protocol for an observational cohort study of Malawian children

**DOI:** 10.12688/wellcomeopenres.19166.1

**Published:** 2023-04-14

**Authors:** Kyle J Wilson, Zhanhan Tu, Emmie Mbale, Priscilla P Mhango, Petros Kayange, Melissa J. Gladstone, Simon Harding, Irene Gottlob, Marta Garcia-Finana, Yaochun Shen, Terrie E Taylor, Karl B Seydel, Yalin Zheng, Nicholas AV Beare

**Affiliations:** 1 Eye & Vision Science, University of Liverpool, Liverpool, England, L69 7TX, UK; 2 Malawi-Liverpool-Wellcome Trust Clinical Research Programme, Blantyre, Southern Region, PO Box 30096, Malawi; 3 Ulverscroft Eye Unit, University of Leicester, Leicester, England, LE2 7LX, UK; 4 Department of Paediatrics, Kamuzu University of Health Sciences, Blantyre, Southern Region, P/Bag 360, Malawi; 5 Department of Ophthalmology, Kamuzu University of Health Sciences, Blantyre, Southern Region, P/Bag 360, Malawi; 6 Women’s and Children’s Health, University of Liverpool, Liverpool, England, L69 7TX, UK; 7 St. Paul's Eye Unit, Royal Liverpool University Hospital, Liverpool, L7 8YA, UK; 8 Cooper Neurological Institute, Cherry Hill, New Jersey, 08002, USA; 9 Department of Health Data Science, University of Liverpool, Liverpool, England, L69 3GF, UK; 10 Department of Electrical Engineering and Electronics, University of Liverpool, Liverpool, England, L69 3GJ, UK; 11 College of Osteopathic Medicine, Michigan State University, East Lansing, Michigan, 4882, USA; 12 Blantyre Malaria Project, Blantyre, Southern Region, P/Bag 360, Malawi; 13 Liverpool Centre for Cardiovascular Science, University of Liverpool, Liverpool, England, L69 7TX, UK

**Keywords:** cerebral malaria, optical coherence tomography, intracranial pressure, brain swelling, artificial intelligence, malarial retinopathy

## Abstract

Cerebral malaria (CM) remains a significant global health challenge with high morbidity and mortality. Malarial retinopathy has been shown to be diagnostically and prognostically significant in the assessment of CM. The major mechanism of death in paediatric CM is brain swelling. Long term morbidity is typically characterised by neurological and neurodevelopmental sequelae. Optical coherence tomography can be used to quantify papilloedema and macular ischaemia, identified as hyperreflectivity.

Here we describe a protocol to test the hypotheses that quantification of optic nerve head swelling using optical coherence tomography can identify severe brain swelling in CM, and that quantification of hyperreflectivity in the macula predicts neurodevelopmental outcomes post-recovery. Additionally, our protocol includes the development of a novel, low-cost, handheld optical coherence tomography machine and artificial intelligence tools to assist in image analysis.

## Introduction

### Background

Despite recent advances in malaria prevention, the statistics remain sobering. In 2021, malaria is estimated to have claimed the lives of 619000 people, with the heaviest burden being felt in sub-Saharan Africa, where 80% of all malaria deaths affect under-fives ^1^. Most of this mortality is due to cerebral malaria (CM) a severe neurological manifestation of the disease. In Malawi, CM is the most common cause of acute coma in the paediatric population.

Sequestration of parasitised red blood cells in the brain microvasculature is the pathological hallmark of CM ^2, 3^. Complex host-parasite interactions result in endothelial dysfunction, inflammatory activation, and dysregulated clotting with microvascular occlusion ^4, 5^. Brain swelling has been implicated as an important mechanism for death in CM, with 84% of children who die having evidence of severe brain swelling on admission MRI ^6^. Recent research suggests that rapid accumulation of microhaemorrhages may contribute to tissue oedema, with transitory uncontrolled fluid outflow to the extravascular compartment with each haemorrhage ^7^.

Given the central role of brain swelling in mortality related to CM, a clinical trial (NCT03300648) is underway in Blantyre, Malawi, to evaluate interventions to reduce intracranial pressure in cases with severe brain swelling (grade 6, 7 or 8) on MRI ^8^. The interventions being trialled against the standard of care are hypertonic saline and mechanical ventilation. Unfortunately, access to MRI in sub-Saharan Africa is extremely limited. Estimates in West Africa suggest there is only one scanner per four million population, with at least half of all scanners in private facilities ^9, 10^.

Papilloedema describes swelling of the optic nerve head (ONH) in cases of raised intracranial pressure (ICP) and is strongly associated with death in CM ^11^. Unfortunately, confident identification of papilloedema is difficult without considerable training, and even then, mild or early cases of papilloedema can be difficult to detect. Furthermore, clinical grading of ONH swelling in papilloedema is limited to the six-point ordinal Frisen scale ^12^ A method for grading papilloedema specific to CM has also been described, but remains limited to an ordinal scale ^13^.

Among survivors of CM, neurological and developmental disabilities (NDD) occur in one third to one half, being evident either at discharge or months after the acute illness ^14, 15^ They include motor and sensory impairments (25%), epilepsy (9%), and cognitive or behavioural impairments (11%). Acute ischaemia from capillary occlusion may be important to the development of NDD ^16^. There is some evidence that parental education, support and skills training improve outcomes for children with acquired disability ^17^.

Optical coherence tomography (OCT) has revolutionised the investigation and management of a range of conditions affecting the retina and optic nerve ^18^. A cross-sectional image of the target structure is created by interpreting interference of low-coherence near-infrared light. Images can be captured through an undilated pupil without contact with the patient’s eye. OCT is a way of detecting swelling of the ONH secondary to raised ICP and can identify ischaemic whitening of the retina in CM ^19, 20^. Descriptive research has shown distinct changes on OCT in CM ^20^.

Advances in artificial-intelligence (AI) approaches to image analysis are rapidly changing the landscape in ophthalmology. AI analysis of OCT images shows great promise in screening and diagnosis of eye disease and is associated with reduced costs when trialled in a clinical environment ^21^.

### Rationale

MRI brain scanning can identify those patients who are most at risk of fatality secondary to severe brain swelling in CM. While the search for suitable treatments for brain swelling in CM is ongoing, MRI is used to identify candidates with severe brain swelling ^8^. Yet in most malaria-endemic areas there is no access to MRI. There is a clear need for a widely available and cost-effective imaging modality to quickly risk stratify children with CM at the bedside. Furthermore, if the test could identify patients at risk of NDD it would facilitate early interventions to educate parents, thus maximising the child’s developmental outcome and strengthening the scientific case for neuroprotective agents in CM.

Here we describe a protocol for a single-site observational cohort study to develop an OCT-based test which identifies severe brain swelling in acute coma, and separately predicts NDD in CM. To make this scalable in the low-and-middle-income countries (LMICs) of malaria-endemic regions we also describe our protocol for the development and validation of a low-cost handheld OCT machine, with AI-enabled algorithms. This will allow practitioners to identify children at risk of death, and those who may benefit from early intervention for NDD without knowledge of OCT image analysis.

### Objectives

Our broad aim is to develop a low-cost OCT-based bedside test with AI analytics to identify and quantify brain swelling in CM and other comas, and to predict NDD post-CM.

Specific research objectives are:

1. To determine the accuracy of OCT-based measures of ONH swelling to detect severe brain swelling in paediatric CM.
2. To evaluate the accuracy of OCT-based measures of macular ischaemia to predict NDD in survivors of paediatric CM at 1 year and 2 years post-recovery.
3. To establish if large accumulation of retinal haemorrhages predicts progression to severe brain swelling in children with CM.
4. To determine the accuracy of OCT-based measures of ONH swelling to detect brain swelling in coma of other cause (COC).
5. To pilot a low-cost handheld OCT equipped with AI analytics and tested to ISO standard and compare useability and effectiveness to a commercially available OCT and human image analysis.

### Study design & location

**Figure 1.**
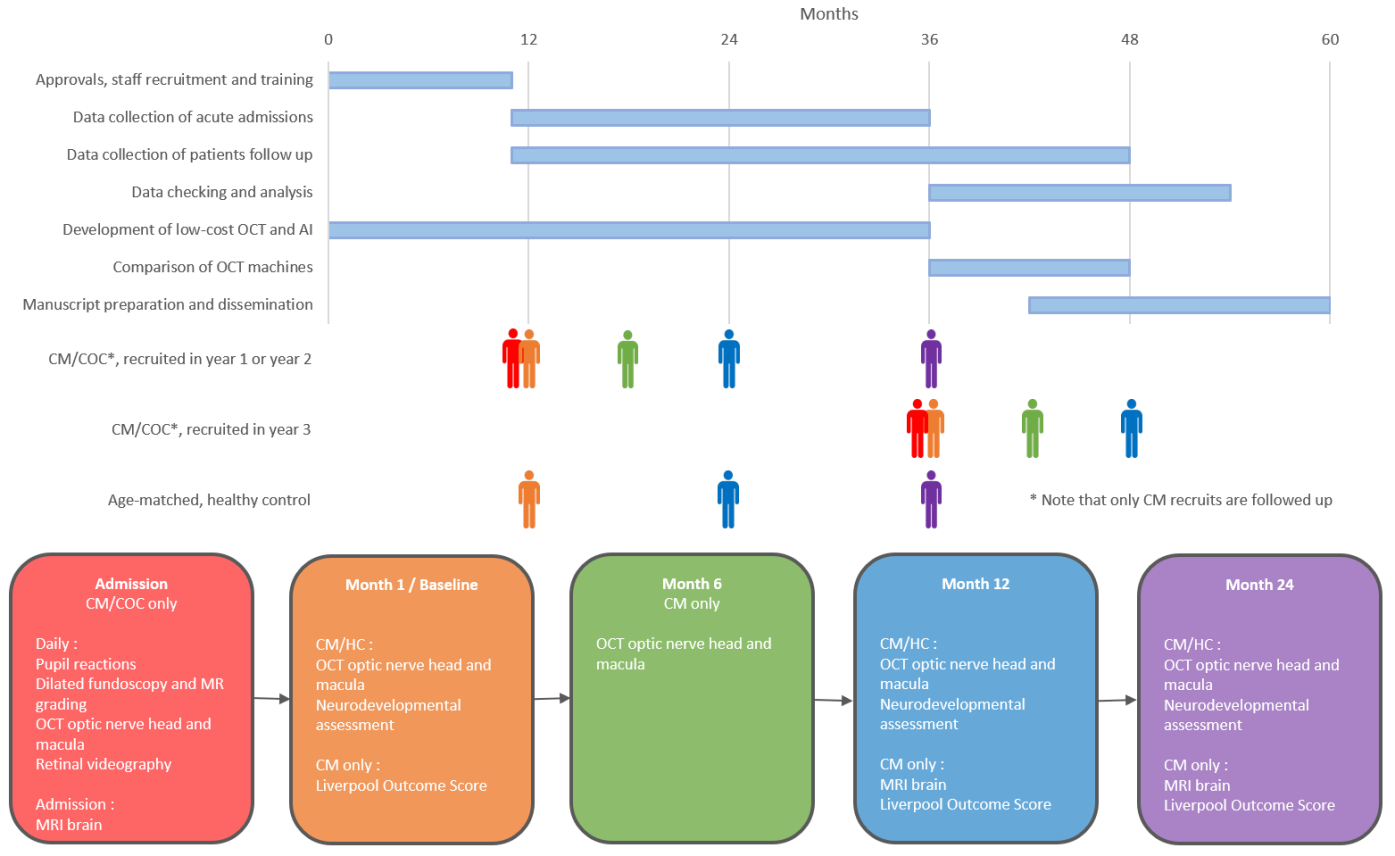
Representative timeline for OCT in CM study and individual participant timelines for different groups. AI – artificial intelligence; CM – cerebral malaria; COC – coma of other cause; HC – healthy controls; MRI – magnetic resonance imaging; OCT – optical coherence tomography

We have designed a single-site observational cohort study of Malawian children. The study will be conducted at the Paediatric Research Ward (PRW) at Queen Elizabeth Central Hospital (QECH), Blantyre, Malawi. The study will aim to recruit 120 patients with CM over a 3-year period, plus a further 90 patients with COC. In addition, we will recruit 60 healthy aged-matched controls, which will augment previously collected data on healthy individuals from the same population. Recruits will be followed up for a period of two years, except those recruited in year three of the study, who will only have one year of follow-up. An illustrative study timeline and the timeline for example participants are shown in Figure 1. Follow-up for the study will augment the standard of care, currently one appointment one-month post-recovery during which the patient is weighed, has a malaria slide, and has a neurological examination performed by a clinician.

### Registration

Trial Registration ISRCTN11735871 ( https://doi.org/10.1186/ISRCTN11735871 registered on 9 ^th^ October 2022).

Protocol Version 4.0: Date 13 ^th^ October 2022.

## Methods

This study is split into three separate work packages. Each work package addresses one or more of our stated research objectives. The objectives associated with each work package are highlighted at the beginning of each of the following sections. Together, all work packages contribute to the broad objective of developing a low-cost OCT-based bedside test with AI analytics to identify and quantify brain swelling in CM and other comas, and to predict NDD post-CM.

### Selection criteria

All admissions to the PRW will be screened according to the following selection criteria. Healthy controls will be invited to attend the PRW where they will be screened against the selection criteria. Participants will be considered eligible if they meet all of the inclusion criteria for a group and none of the exclusion criteria.

***Work Package 1***

Inclusion criteria (CM group):

1. Parent or guardian gives informed consent for participation
2. Aged under 16 years
3. Blantyre Coma Score ≤ 2
4. Peripheral parasitaemia or positive malaria rapid diagnostic test
5. No other cause for coma evident

Inclusion criteria (COC group):

1. Parent or guardian gives informed consent for participation
2. Aged under 16 years
3. Blantyre Coma Score ≤ 3
4. No peripheral parasitaemia and negative malaria rapid diagnostic test

Exclusion criteria (CM / COC groups):

1. Hypoglycaemia, post-ictal or other transient state accounting for coma
2. Contraindication to MRI
3. History or evidence of pre-existing hydrocephalus, significant neurological disease, neurological disability or learning difficulties
4. History or evidence of pre-existing significant ocular disease
5. History or evidence of severe life limiting illness, including but not limited to advanced HIV/AIDS (Grade 4), disseminated TB and malignancy

Inclusion criteria (healthy controls):

1. Parent or guardian gives informed consent for participation
2. Aged under 16 years

Exclusion criteria (healthy controls):

1. History or evidence of pre-existing hydrocephalus, significant neurological disease, neurological disability or learning difficulties
2. History or evidence of pre-existing significant ocular disease
3. History or evidence of severe life limiting illness, including but not limited to advanced HIV/AIDS (Grade 4), disseminated TB and malignancy

***Work Package 2***

All patients eligible to participate in WP1 by meeting the criteria for the CM group or the healthy control group will be eligible for WP2 and will be automatically enrolled to WP2.

***Work Package 3***

All patients eligible to participate in WP1 or WP2 recruited during year 3 or year 4 of the study will be eligible for WP3 and will be automatically enrolled to WP3.

### Work Package 1 (WP1) – Procedure and Outcomes

This work package addresses objectives 1, 3 and 4. The study population is children under 16 years with coma secondary to CM or another cause. The anticipated sample size is 120 children with CM and 90 with COC. This represents the expected number of COC admissions in the time required to recruit 120 patients with CM. For details of sample size calculations, see section ‘Sample Size & Statistics’.

Enrolled patients will have their pupillary reflexes tested and recorded, before having both eyes dilated with Tropicamide 1% and Phenylephrine 2.5%. Once adequately dilated patients will have both eyes examined with direct and indirect ophthalmoscopy with standardised grading of malarial retinopathy (MR) ^22^. OCT scans of the ONH and macula will be acquired from both eyes using the Leica Envisu C2300 handheld OCT machine (Leica Microsystems, Germany) by an ophthalmologist experienced in the use of handheld OCT or one of two research nurses who have undergone specific training in acquisition of OCT scans. This training included obtaining OCT scans on healthy adults with direction from an experienced ophthalmologist, and conducting OCT scans in healthy children with the same ophthalmologist providing feedback. Retinal video of both eyes will be captured using a Epicam M handheld retinal camera (Epipole, UK) to accurately enumerate the number of retinal haemorrhages. This will be conducted by an ophthalmologist or an imaging technician experienced in the use of the Epicam M. Full details of these procedures are available in the Standard Operating Procedures. Patients will also have an admission MRI on the Paediatric Research Ward using the 0.064T Hyperfine Swoop MRI scanner (Hyperfine Research Inc, UK), or if available the 0.35T MRI scanner (GE Healthcare, USA) in the local radiology department. MRI protocols will match those used by the Treating Brain Swelling study (NCT03300648) ^8^. Fundus videography and OCT will be repeated daily while patients remain in hospital and cooperative.

The test under evaluation is ONH OCT as a surrogate marker of brain swelling. The gold standard test is MRI brain graded for swelling on an eight-point scale by an experienced radiologist. We will prospectively analyse ONH parameters including ONH volume, retinal nerve fibre layer thickness and cup:disc ratio to assess the area under the curve (AUC) for identifying severe brain swelling in CM. A similar analysis will be undertaken for COC. We will also enumerate the haemorrhages using the retinal videos. The test under investigation is absolute number and change in number of retinal haemorrhages as a marker of progression to severe brain swelling. This will be analysed in relation to admission MRI findings, findings from any repeat MRIs (either clinically indicated or obtained as part of a parallel study) and outcome. Retinal haemorrhages will be considered both independently of and alongside ONH OCT.

### Work Package 2 (WP2) – Procedure and Outcomes

This work package addresses objective 2. The study population is children under 16 years with coma secondary to CM. The anticipated sample size is 120 children with CM, plus 60 healthy, age-matched controls. In addition to OHN OCT for WP1, enrolled CM patients will have OCT macula performed to try to identify retinal ischaemia, which is visible as hyperreflectivity on OCT. Areas of hyperreflectivity will be identified and quantified following manual segmentation.

CM patients will return one-month post-discharge for repeat OCT and neurodevelopmental assessments. The neurodevelopmental assessment will consist of the Malawi Developmental Assessment Tool (MDAT) for children under five years of age, Kaufman Assessment Battery for Children (K-ABC) for those older than five years of age, as well as the Liverpool Outcome Score for all children. All have been validated in Africa ^23– 25^. From the results we will create an age-adjusted measure of global development and cognition. Follow-up at 6 months (OCT only), 12 months and 24 months (OCT, MRI, and neurodevelopmental assessment) will provide longitudinal data, with primary outcome assessed at month 12.

Age-matched controls will be recruited from the community, siblings of recruits and children with acute non-neurological illness presenting to QECH. They will have a medical and developmental history taken, pupil dilation, fundoscopy, OCT of ONH and macula, and neurodevelopmental assessment at baseline, 12 months, and 24 months. The neurocognitive status of healthy controls will help to define neurocognitive deficit in CM cases. Neurocognitive data on our 60 controls will be added to existing data on 100 healthy subjects from the same population and case z-scores determined. Two z-scores below the mean defines abnormal. This data has been generated from studies ongoing at the Blantyre Malaria Project with well-established eligibility criteria and protocols consistent with our own.

### Work Package 3 (WP3) – Procedure and Outcomes

This work package addresses objective 5. The development of a novel, low-cost, handheld OCT machine will be based on our current table-mounted prototype, designed for screening of diabetic retinopathy in China. Development will follow a medical device design approach, complying with regulatory requirements. Specifically, design and development processes will be accompanied by risk management activities according to International Standards Agency (ISO) 14971. Accompanying Class B software will be constructed to International Electrotechnical Commission (IEC) 62304:2006 requirements.

Novelties of the proposed device include: a fibreoptics-based spectrometer and novel microelectromechanical systems with integrated drivers for robustness and compactness, targeted to applications in low-resource settings; novel optics design, providing a greater field of view and greater engineering tolerances, thereby reducing production costs and facilitating maintenance; optional integration with a scanning laser true-colour fundus camera, developed by our group for diabetic retinopathy screening.

Prior to deployment the device will undergo rigorous laboratory testing. First, testing will ensure that light power output is within recognised safety standards (ISO15004-2, light safety standard for ophthalmic instruments). We will also ensure the proposed device achieved class 1 standard when assessed against IEC 60825-1. The internal laser will be certified by both an internal (University of Liverpool) and a certified external laser safety expert. As the device will be deployed in Malawi, it will also be constructed to any standards set by the Pharmacy and Medicines Regulatory Authority, to whom responsibility for medical devices in Malawi is delegated, and to the requirements of the Malawi College of Medicine Research Ethics Committee.

Finally, the novel handheld OCT will be evaluated against the commercially available Leica Envisu C2300 handheld OCT (Leica Microsystems, Germany). The study population will be all participants recruited to WP1 or WP2 in years 3 and/or 4 of the study. OCT of the ONH and macula will be performed with both devices in alternating sequence. Operators will ensure that the corneas remain lubricated during the obtaining of OCT scans with both devices by manually closing the upper eyelid or use of lubricating eye drops. The operator will record ease of use on a five-point scale, time taken to acquire OCT scans and any factors affecting scan quality. At image analysis a quality assessment of the images will be made (good, acceptable, unacceptable/ungradable). Measured parameters from the optic nerve head and extracted data on hyperreflectivity and other parameters in the macula will be compared between the two devices using intraclass correlation coefficients. Kappa statistics (and other comparative statistics) will be used to compare the human image analysis with the computerised AI analysis. This will be done according to the Standards for Reporting Diagnostic Accuracy Studies–Artificial Intelligence if available (currently under development). 

This part of WP3 is limited in scope to piloting the novel OCT’s useability and effectiveness, comparing to the commercially available machine. It is not conducted with a view to commercialisation of the novel OCT. If the results are positive then a further multicentre clinical study would be planned, with new protocols and approvals, with a view to more widespread assessment and implementation.

### Follow-Up

Parents/guardians of participants will have the study explained to them in detail during the consent process and will be invited for follow-up. Their telephone number and a verbal description of where they live will be recorded. Where participants fail to attend follow-up their parent/guardian will be contacted by telephone. If this fails a clinical officer will visit them at home and invite them to attend.

Subjects (over the age of assent, or else their parents or guardians) can withdraw from the study at any time, for any reason. It is not required, but should they choose to provide the reason it will be recorded.

### Sample size and statistical analysis

The study sample size calculation is based on the primary research question: determining the accuracy of OCT measures of ONH swelling to detect severe brain swelling in children with CM.

A diagnostic test’s optimal specificity/sensitivity can be based on its receiver operating curve (ROC) with an optimal AUC being close to 1 ^26^. For our primary analysis (to differentiate severe from non-severe brain swelling in CM), the minimum sample size was calculated with an estimate of the AUC of 0.8 aiming to achieve 95% confidence with half-width of the confidence interval of ±0.07 ^27^. The anticipated AUC is based on preliminary data from Blantyre, and the ratio of severe:non-severe brain swelling cases from published data ^6^. For an acceptable sensitivity of 90% and specificity of 80%, 120 cases are required (42 with severe brain swelling, 78 children with non-severe brain swelling).

As all recruited patients will be acute admissions to hospitals, and the primary outcome measure (the binary variable representing severe or non-severe/no brain swelling) will be recorded during their hospital stay, we have not included any loss-to-follow up in this calculation.

For objectives 1 and 4 the ROC will be calculated for several ONH parameters including ONH volume, retinal nerve fibre layer thickness and cup:disc ratio to determine which parameter or combination of parameters has the best AUC for identifying severe brain swelling in CM. 

For objective 2, the association between the extent of macular hyperreflectivity on OCT and severity of NDD will be assessed, including new epilepsy, neurological impairment, developmental delay, and behavioural problems. The ability of macular hyperreflectivity to predict NDD will be investigated with an adjusted regression model.

Objective 3 is exploratory, seeking to determine if an increase in retinal haemorrhages is associated with the development of severe brain swelling, with or without data from ONH OCT. The association between the number of haemorrhages on admission and change in haemorrhages over time will be assessed against progression in MRI-detected brain swelling. This will be assessed in a subgroup of patients who have two or more MRI scans. ONH parameters will be factored in to see if the addition of ONH data increases the predictive value of retinal haemorrhages.

To evaluate objective 5, we will compare the observer scores for the two OCT machines using Wilcoxon signed-rank test for paired ordinal data. Image quality will be assessed similarly. Measured parameters from the optic nerve head and extracted data on hyperreflectivity and other parameters in the macula will be compared between the two devices using intraclass correlation coefficients. Kappa statistics (and other comparative statistics) will be used to compare the human image analysis with the computerised AI analysis.

### Consent & ethics

This study will be conducted in accordance with the ethical principles as outlined in the Declaration of Helsinki and in accordance with Good Clinical Practice (GCP) guidelines.

Written, informed consent will be sought from the parent/guardian of all participants once the patient’s condition has stabilised, or upon arrival for healthy controls, by trained research nurses fluent in both English and Chichewa. For children over the age of 12 years assent will be sought in accordance with local procedures and ethical requirements. Participant information sheets are available in English and Chichewa. Unconscious children are potentially vulnerable and accordingly Blantyre Malaria Project have conducted recent qualitative studies with parents/guardians to better understand the challenges of obtaining informed consent in a paediatric critical care setting in LMICs ^28^. Only participants who are freely consenting will be considered eligible for the study. Patients will then be screened according to the inclusion and exclusion criteria before being enrolled.

This protocol describes as observational study and will not influence patient care or enrolment into interventional studies in any way. Ocular examination and imaging will only be initiated once the clinician in charge is happy that the patient is sufficiently stable and once treatment has been initiated. Pupil dilation and fundoscopy are already part of standard care on the PRW. OCT scans are performed at the bedside with a handheld, non-contact device. Retinal video is captured for approximately 2 minutes per eye with the EpiCam fundus video camera using visible light. Together, ocular examination and imaging are anticipated to take approximately 30–45 minutes. Chloral hydrate will be used to induce sleep in children attending for MRI scans at follow-up. This is routine practice in studies involving MRI at the Blantyre Malaria Project as it reduces movement artefact, aiding acquisition of high-quality images in young children. The procedure is low risk and is explained to parents during the consent process.

Healthy controls will be sought from the community, siblings of recruits and children with acute non-neurological illness presenting to QECH. Prospective block-matching will be used to age-match controls. Conscious children tolerate the light and instrumentation of OCT scans well in pilot studies conducted in Blantyre. Fundoscopy requires a brighter light, but sufficient examination can usually be performed with patience.

Both CM cases and controls will undergo three neurodevelopmental testing sessions over 2 years in the presence of their parent/guardian. It is expected that some CM cases will be identified as having a neurodevelopmental delay or disorder, and it is possible that some control children might also. These children will be referred to appropriate clinical services provided by community and paediatric units. Blantyre Malaria Project already has these referral pipelines in place through previous projects and we will utilise these networks and relationships to ensure provision of timely and effective interventions to children identified to be in need. We will also offer a counselling session for parents with children identified with a developmental disorder. The session will cover four main points: 1) getting children engaged, 2) preventing challenging behaviours, 3) teaching new skills, and 4) helping children with motor problems.

Parents/guardians of both controls and cases will be reimbursed for time and travel for attending study visits in accordance with guidance from Kamuzu University of Health Sciences, Blantyre, Malawi.

This study has ethical approvals from the University of Liverpool Research Ethics Committee (11225, 28 ^th^ June 2022), Liverpool, UK, the College of Medicine Research and Ethics Committee (P.11/21/3460, 2 ^nd^ June 2022), Kamuzu University of Health Sciences, Blantyre, Malawi, and Michigan State University Institutional Review Board (STUDY00007131, 28 ^th^ March 2022), East Lansing, Michigan, USA.

The study is sponsored by the University of Liverpool (Reference UoL001660).

sponsor@liverpool.ac.uk

Clinical Directorate

4th Floor Thompson Yates Building

Faculty of Health and Life Sciences

University of Liverpool

Liverpool

L69 3GB.

### Study oversight

The study will have an Oversight Committee of independent persons, including a Malawian clinician and lay person. The funder and sponsor will be observing members of this committee. As this is an observational study there is no Data Monitoring Committee planned. The Malawi-Liverpool-Wellcome Trust Clinical Research Programme’s Clinical Research Support Unit will undertake study monitoring and ensure compliance with governance and data quality requirements. They will conduct annual study review and full audit if deemed necessary, which will be independent from investigators and the sponsor.

### Data management

All clinical and laboratory data will be recorded in the CRF and stored with a unique patient identifier. Data will be entered onto a REDCap database by double data entry. Where appropriate automatic range checks are included for numerical data in the REDCap entry forms. All data will be backed up and backup copies stored on site and in Liverpool. Data transfer will be managed with a secure internet connection or using an encrypted hard drive. Paper records will be stored in locked cabinets. Access to paper records will be limited to study personnel with prior authorisation (by the site principal investigator). All data will be pseudo-anonymised prior to presentation or publication of any results. Participants will be identified by a unique patient identifier only. Patient identifiable information will not be recorded on the study database in compliance with GCP requirements. The data will be examined for inconsistencies during the trial by the clinical research fellow. Inconsistencies will be discussed with the chief investigator prior to any corrections. Corrections will follow GCP procedures.

### Confidentiality

All clinical data will be held confidentially in accordance with GCP guidance. Personal identifiers will be removed before analysis of the data and dissemination of the results.

### Data sharing

Study data linked to participants (pseudo-anonymised) will be kept for a maximum of five years and anonymised data will be kept for a minimum of ten years. Data will be archived electronically on MLW and UoL servers. After completion of the study researchers may request access to the pseudo-anonymised study data from the chief investigator who will discuss the request the clinical research support unit. Researchers may be required to provide a study protocol and plan for secondary data analysis. Data collection tools and standard operating procedures are available upon reasonable request to the chief investigator.

Data will be shared with collaborating authors subject to the terms of institutional contracts.

### Protocol amendments

Protocol or standard operating procedure amendments will be communicated with the relevant ethics committees and review boards electronically prior to their implementation. If a safety risk is identified then recruitment will be paused while protocol amendments are subject to review by relevant committees. Updated protocols or standard operating procedures will be updated in the Investigator Site File in accordance with GCP guidance.

### Dissemination of results

The results of this trial will be published in an open-access journal in accordance with funder and institutional requirements. Requirements for authorship will confirm to the ‘Uniform Requirements for Manuscripts Submitted to Biomedical Journals’ as developed by the International Committee of Medical Journal Editors. Interim analyses will be shared at appropriate academic meetings. Findings deemed important for participants will be communicated with them at follow-up or by telephone on request. The public engagement team at Malawi-Liverpool-Wellcome Trust will facilitate dissemination via radio of significant findings which affect the local population.

A version of the full protocol prepared for publication will be published in a peer-reviewed open access journal. A version prepared for the funder is available at the ISRCTN registry ISRCTN11735871 ( https://doi.org/10.1186/ISRCTN11735871). Code for statistical analyses will be shared as supplementary material for any accepted publications, if permitted by the accepting journal. If not permitted code will be made available upon reasonable request. For details of data sharing see ‘Data Sharing’.

### Monitoring

The Clinical Research Support Unit at Malawi-Liverpool-Wellcome Trust will be responsible for monitoring and governance during the trial. This will include interim review of the trial’s progress, including updated figures on recruitment, data quality, adherence to trial protocols and monitoring of adverse events. The Clinical Research Support Unit is supported by a core grant from the funder and are paid a contribution from Grant Number: 222530/Z/21/Z for their services.

## Conclusions

This clinical study will further the understanding of the pathogenesis of CM, building on previous critical insights from the study of MR. Success in this programme will lead to multicentre trials utilising OCT across the International Centres of Excellence in Malaria Research.

A non-invasive bedside test for rapidly identifying brain swelling in children may be applicable in other neurological diseases. Similarly, the ability to identify CNS ischaemia, and the development of methods for quantifying retinal ischaemia and correlating this to the degree of NDD in CM, may carve a path for similar research into other causes of NDD in children.

Development of a low-cost, handheld OCT machine with demonstrable utility in low-resource settings will lead to further research to develop the machine for widespread use with a view to training of technicians in LMICs to be able to quickly add prognostic information in CM and other comas.

## Study status

This study is currently in the recruitment phase.

## Data Availability

Zenodo. SPIRIT Checklist & Model Consent for 'Predicting Acute and Post-Recovery Outcomes in Cerebral Malaria and Other Comas by Optical Coherence Tomography (OCT in CM) – A protocol for an observational cohort study of Malawian children'. DOI: https://doi.org/10.5281/zenodo.7703643 ^29.^ This dataset contains the SPIRIT checklist (adapted to an observational trial) and model consent forms for the OCT in CM study protocol. The protocol will be submitted as a paper to Wellcome Open Research. Data are available under the terms of the Creative Commons Zero "No rights reserved" data waiver (CC BY 4.0 Public domain dedication).
