# Supplementary material for: Predicting Acute and Post-Recovery Outcomes in Cerebral Malaria and Other Comas by Optical Coherence Tomography (OCT in CM) – A protocol for an observational cohort study of Malawian children
Source: Wellcome Open Res. 2023 Apr 14;8:172. [Version 1] doi: 10.12688/wellcomeopenres.19166.1 (PMC10468659; doi:10.12688/wellcomeopenres.19166.1)
Supplement: Peer review history

# Peer review: Reviewer response for version 1

**DOI:** 10.21956/wellcomeopenres.21246.r63453

**Published:** 2023-08-30

**Authors:** Monique F Stins

**Affiliations:** 1 Johns Hopkins School of Public Health, Baltimore, MD, USA

Cerebral malaria (CM) has a high mortality and about one third of the survivors is left with neurological and developmental disabilities.  CM can lead to brain swelling which eventually can contribute to the death of the patients. Several biomarkers to assess the severity of CM are available, including fundoscopy, which also can distinguish between real CM and other factors contributing to the coma and severity of CM. However, a good, affordable/low-cost and non-invasive method to assess the degree of brain swelling, to predict the outcome of CM and chance of full recovery is not yet available, especially in LMIC settings.

 Researchers propose using Optical coherence tomography (OCT) to assess the degree of optic nerve swelling (papiloedema) as a measure for brain swelling in CM patients. As OCT instrumentation is relatively large and expensive, it is proposed to develop a new low cost hand-held OCT model. In addition, an AI protocol will be developed to aid with the interpretation of the data/image analysis.

 OCT can be a great and effective tool to investigate swelling of the optic nerve. How much the swelling of the optic nerve relates to brain swelling in CM is not clear. Researchers propose to do this by a comparison with MRI, which is available in their research hospital. The timing of the assessments can is critical.

 MRI can be done with sick and comatose patients, however, when the patient is not alert, doing an OCT is challenging. The work packages indicate children <16 years with coma, with the presence of coma, the assessments, especially in small children, will be challenging. However, researchers have experience with this, as published before (Tu et al 2121) and they are building further on this expertise.  Training of the local nurses has been done on healthy adults and children (age?), but as currently listed, not on comatose patients and children yet. This will be challenging.

 All the listed research objectives; determine accuracy in relation to 1) brain swelling, 2) macular ischemia, 3) potential other causes, 4) relation of retinal haemorrhages to brain swelling and 5) to pilot a low-cost hand-held OCT, are valid. The availability of this data would significantly add to the evaluation and progress of the infection, and prediction of outcome.

 Follow up (2) to predict status of survivors is proposed to be 1 and 2 year post infection.

 MRI is done at admission, at 1 and 2 years. This long term follow-up will be useful to indicate degree of recovery versus sequelae.

 The usability and effectiveness of the novel OCT’s  will be compared to a commercially available machine. With positive results a follow up study will be done. Although researchers indicate that this is not done with the purpose of commercialization, in the end this is usually a component of any interested entity/manufacturer. In this light, some discussion on making this available for an affordable price in LMIC settings would be in place.

 Inclusion/ exclusion criteria are valid/reasonable.

 A loss to follow up is not taken into account. I assume investigators expect this to be low and that the remaining data will be sufficient to analyze. However, a discussion of the expected loss with respect to statistical analysis should be done.

 It is stated that neurological assessment will be done and behavioral problems tracked. Which neuro assessments will be included, how is this broken down into age groups and how will the behavioral assessments be tracked/ recorded? This would need some more clarification.

 Its is very good to see that counselling session for patients with developmental disorders will be done.

 What is the current status of the hand-held OCT? Based on the previous publication of this group, the development may be further along than stated in the application (at time of submission). Therefore, earlier results than the proposed 3 years to develop this could be possible. Thus, higher number of patients could be potentially tested for the comparisons.

Is the study design appropriate for the research question?

Yes

Is the rationale for, and objectives of, the study clearly described?

Yes

Are sufficient details of the methods provided to allow replication by others?

Yes

Are the datasets clearly presented in a useable and accessible format?

Not applicable

Reviewer Expertise:

NA

I confirm that I have read this submission and believe that I have an appropriate level of expertise to confirm that it is of an acceptable scientific standard.

# Peer review: Reviewer response for version 1

**DOI:** 10.21956/wellcomeopenres.21246.r58802

**Published:** 2023-07-14

**Authors:** Leonardo J M Carvalho

**Affiliations:** 1 University of Campinas, Campinas, Brazil

This protocol by Wilson and colleagues is designed, as per the authors, “to test the hypotheses that quantification of optic nerve head swelling using optical coherence tomography can identify severe brain swelling in CM, and that quantification of hyperreflectivity in the macula predicts neurodevelopmental outcomes post-recovery. Additionally, our protocol includes the development of a novel, low-cost, handheld optical coherence tomography machine and artificial intelligence tools to assist in image analysis”.

 The protocol is well designed and very much welcome, as optical coherence tomography (OCT) has a great potential as a simple, non-invasive and informative tool for assessing and staging brain swelling and other pathological consequences of cerebral malaria and, therefore, can be a critical aid in diagnosis, treatment and prevention/management of post-CM neurological sequelae. The protocol is also designed to develop a novel low-cost OCT equipment, which has the potential to facilitate adoption of this important tool in low resource settings.

 The rationale for, and objectives of, the study are clearly described, and work packages 1, 2 and 3 provide a clear description of how the objectives will be addressed and attained. If objectives are completed and OCT can reliably mirror MRI findings to identify brain swelling and “quickly risk stratify children with CM at the bedside”, this study can have a big impact in CM patient management in low resource areas, helping saving lives and improving quality of life in post-CM recovery.

 The study design is appropriate for the research question. As described, it is “a single-site observational cohort study to develop an OCT-based test which identifies severe brain swelling in acute coma, and separately predicts neurological and developmental disabilities in CM”.

 The protocol provides sufficient details of the methods to allow replication by others, and plans for data management and data sharing are described.

 One doubt arises as the study sample size calculation is based on the primary research question: determining the accuracy of OCT measures of ONH swelling to detect severe brain swelling in children with CM. It is not clear whether this sample size is also adequate to attain objective 2 (the association between the extent of macular hyperreflectivity on OCT and severity of NDD), especially considering that this involves a 2-year follow up.

Is the study design appropriate for the research question?

Yes

Is the rationale for, and objectives of, the study clearly described?

Yes

Are sufficient details of the methods provided to allow replication by others?

Yes

Are the datasets clearly presented in a useable and accessible format?

Yes

Reviewer Expertise:

Malaria

I confirm that I have read this submission and believe that I have an appropriate level of expertise to confirm that it is of an acceptable scientific standard.
